# Improving estimation of puma (*Puma concolor*) population density: clustered camera-trapping, telemetry data, and generalized spatial mark-resight models

**DOI:** 10.1038/s41598-019-40926-7

**Published:** 2019-03-14

**Authors:** Sean M. Murphy, David T. Wilckens, Ben C. Augustine, Mark A. Peyton, Glenn C. Harper

**Affiliations:** 1 Wildlife Management Division, New Mexico Department of Game & Fish, Santa Fe, 87507 USA; 2 Atkinson Center for a Sustainable Future, Department of Natural Resources, Cornell University, Ithaca, 14853 USA; 3 Valles Caldera National Preserve, U.S. National Park Service, Jemez Springs, 87025 USA; 4 Department of Natural Resources, Pueblo of Santa Ana, Santa Ana Pueblo, 87004 USA; 5 Present Address: Department of Forestry and Natural Resources, University of Kentucky, Lexington, 40546 USA

## Abstract

Obtaining reliable population density estimates for pumas (*Puma concolor*) and other cryptic, wide-ranging large carnivores is challenging. Recent advancements in spatially explicit capture-recapture models have facilitated development of novel survey approaches, such as clustered sampling designs, which can provide reliable density estimation for expansive areas with reduced effort. We applied clustered sampling to camera-traps to detect marked (collared) and unmarked pumas, and used generalized spatial mark-resight (SMR) models to estimate puma population density across 15,314 km^2^ in the southwestern USA. Generalized SMR models outperformed conventional SMR models. Integrating telemetry data from collars on marked pumas with detection data from camera-traps substantially improved density estimates by informing cryptic activity (home range) center transiency and improving estimation of the SMR home range parameter. Modeling sex of unmarked pumas as a partially identifying categorical covariate further improved estimates. Our density estimates (0.84–1.65 puma/100 km^2^) were generally more precise (CV = 0.24–0.31) than spatially explicit estimates produced from other puma sampling methods, including biopsy darting, scat detection dogs, and regular camera-trapping. This study provides an illustrative example of the effectiveness and flexibility of our combined sampling and analytical approach for reliably estimating density of pumas and other wildlife across geographically expansive areas.

## Introduction

Pumas (cougars or mountain lions; *Puma concolor*) are the most widely distributed large carnivore in the western hemisphere^1^. Similar to other large carnivores, pumas have considerable resource requirements and provide important ecological benefits over expansive areas^1–3^. Their presence sometimes results in conflicts with humans, however, and predation by pumas can influence vital rates of terrestrial ungulate populations^4,5^. Although some puma populations have recently expanded range and present novel management challenges^6,7^, other populations are small, isolated, or otherwise imperiled and might necessitate conservation intervention^8,9^. Conservation and management of pumas are often contentious issues that are influenced by multiple political, social, and economic interest groups, and resolving disputes has increasingly hinged on managing authorities possessing reliable and contemporary estimates of puma population density and abundance^10–12^. However, pumas are wide-ranging, cryptic, and notoriously difficult to detect; consequently, few jurisdictions within the species’ occupied range have reliable estimates of those demographic parameters. Most puma populations are instead managed based on population indices, such as hunter effort, mortality trends, or expert opinion, extrapolation of densities from small study areas and other jurisdictions, or a combination thereof^10,13–15^, all of which may be unreliable and could result in flawed conservation and management^16,17^.

Spatially explicit capture-recapture models integrate a detection process model with an ecological process model that describes the spatial distribution of animal activity centers, or home range centers, across a study area, and can produce unbiased estimates of population density^18,19^. Recent studies have applied spatially explicit models to multiple types of detection data to estimate puma population density; for example, tissue samples collected by biopsy darting pumas that were treed using hounds^20–22^, puma scat collected via area searches by scat detection dogs^23^, and photographs of pumas collected from regular or contiguous arrays of remote camera-traps^24–27^. However, biopsy darting and scat detection dog sampling necessitate often expensive laboratory genetic analyses to produce individual identities from detection data^28^. Additionally, treeing pumas with hounds for biopsy darting is likely most efficient during winter and in locales with sufficient snow cover that improves tracking^20,22^, and because of high DNA degradation rates in scat that can reduce sample sizes, optimal effectiveness of scat detection dog sampling is generally limited to locales with cool and dry climates^29,30^. In contrast, remote camera-trapping can be a cost-efficient and logistically feasible approach for effectively detecting pumas and other large carnivores across habitats, ecosystems, and climatic conditions^31,32^.

A critical assumption of most capture-recapture models is that all detected animals are individually identifiable^19^. This can be difficult to achieve if camera-traps are used to detect pumas or other wildlife that lack visible, individually unique natural markings, such as the rosettes on jaguars (*Panthera onca*)^24,33^. To overcome this issue, mark-resight models and their spatially explicit analogues, spatial mark-resight (SMR) models, were developed to estimate the density of populations in which only a portion of animals are individually identifiable^26,34–37^. Attempting to assign individual identities to pumas *ad hoc* based on perceived natural marks, such as scars, ear nicks, body shapes, or carriages^25,27^, can result in biased and unreliable density estimates, however, because multiple individuals may have similar physical features, causing observers to agree on incorrect identity assignments or disagree on correct identity assignments^24^. Furthermore, given the ambiguity, it is not always possible to identify a sufficient number of individually unique pumas based solely on natural marks to estimate population density^24,38^.

For pumas and other species that lack unambiguous natural markings, physically capturing and applying artificial marks, such as radiocollars or ear tags, to a portion of animals in a population is likely necessary for accurate density estimation when using camera-traps for detection^26,32,34–37^. Such mark-resight methods can be viable, cost-effective alternatives to capture-recapture methods, because only a single marking event of a portion of a population is required and camera-trapping to collect resighting data is efficient. Using Global Positioning Systems (GPS) collars as marks can permit unambiguous individual identification for nearly all camera-trap detections of marked individuals, assist with determining whether an animal is marked or unmarked, and also provide telemetry location data that can be integrated in spatially explicit models to improve estimation of individual activity centers, the detection function spatial scale (home range) parameter (σ), and ultimately, population density^26,36,37,39^.

One challenge associated with using researcher-applied artificial marks is that in SMR models, the spatial distributions of marked and unmarked individuals across the landscape are informed by the capture and marking process; therefore, correctly specifying those distributions in the process model is critical for accurately estimating population density^35,37^. Conventional spatial mark-resight (conSMR) models assume that marked and unmarked individuals have the same spatial distribution, typically uniformity or that the two distributions can be specified correctly with parametric distributions^26,34,36^. Although the assumption of spatial uniformity may be valid for jaguars and other species that are identifiable by their individually unique natural markings, it is likely inappropriate if animals are physically captured and artificially marked, because of the juxtaposition between marking and resighting locations^35,37^. If the marking and resighting detector arrays overlap, animals that are captured for marking are located on average closer to the resighting array than unmarked individuals and, therefore, likely will have higher detection rates than unmarked individuals. Consequently, if researcher-applied artificial marks are used for individual identification, conSMR models, which do not account for the capture and marking process, may underestimate the numbers of both unmarked and undetected individuals and thus, population density^35,37^.

A generalized spatial mark-resight model (genSMR) was recently developed that resolves this problem by including sub-models for both the marking and resighting processes^37^. This allows the differing spatial distributions of marked and unmarked individuals to be determined by the marking process, and simulations have demonstrated that the genSMR model produces unbiased estimates of population density when marking is not random across a study area^37^. The parameters of the genSMR model developed by Whittington *et al*.^37^ are estimated via Bayesian methods using Markov chain Monte Carlo (MCMC) algorithms. In contrast, Efford and Hunter^35^ developed a pseudolikelihood-based model and estimation procedure that is analogous to genSMR, which they refer to as spatial capture-mark-resight. A primary limitation of this pseudolikelihood estimation procedure is that it ignores information contained in the spatial distribution of detections of unmarked individuals. Efford and Hunter^35^ argued that the information lost by discarding these data is minimal; however, the magnitude of information in the spatial locations of detections of unmarked animals can be increased through the use of partial identity covariates^34,39^.

A key source of uncertainty in SMR models stems from the need to probabilistically resolve the individual identities for detections of unmarked animals, as well as detections of marked but unidentifiable animals and animals with unknown mark status, if available^34,39^. Reducing uncertainty in the individual identity assignments can reduce the uncertainty in population density estimates, which can be accomplished with partial identity covariates^39,40^. The use of categorical partial identity covariates in the form of microsatellite loci genotypes has been demonstrated^39,40^, but the utility of partially identifying information in camera-trap studies, where animal sex and other potential covariates are fewer in number and less reliably determined from photographs, has not been explored. Such covariates are typically either not recorded or are discarded from camera-trap detection data, so evaluating their effectiveness for improving the precision of parameter estimates from spatially explicit models could result in improved density estimation in camera-trapping studies.

Because of the logistical and financial constraints associated with currently available puma sampling methods and survey designs, researchers are often forced to estimate puma population density for areas that are smaller than the geographical extent of populations or the scale at which conservation and management occur^10,15^. Population density estimates are then extrapolated to larger areas, typically with considerable uncertainty and unverified assumptions^10,13–15^. By incorporating spatial information about when and where individual animals are detected, spatially explicit models are robust to irregular sampling designs, such as clusters of detectors with gaps between clusters, which can permit efficient surveying of large geographical areas^18,41–45^. Recent studies evaluated clustered sampling designs of noninvasive genetic hair-traps in the spatially explicit framework for estimating American black bear (*Ursus americanus*) population density, which demonstrated that density estimates were improved, largely because more individuals were exposed to detectors and spatial recaptures were obtained over expansive areas^41,43–45^. Remote camera-trapping is arguably the most widely used and practical noninvasive method for surveying wildlife populations globally^31,32^; therefore, considerable potential exists for using clustered sampling designs in camera-trap studies to estimate population density over spatially extensive areas, which could have widespread practical utility across terrestrial wildlife species and geographical locales.

Herein, we apply clustered sampling to camera-traps in the spatially explicit framework to demonstrate the potential for this approach to survey pumas over expansive areas with reduced effort. We then apply recently developed genSMR models to the obtained camera-trap detection data to estimate puma population density and abundance. In addition, we evaluate the influence on parameter estimates of integrating telemetry data from GPS collars on marked pumas, incorporating sex as a categorical identity covariate for unmarked pumas, and accommodating activity center transiency. Our results demonstrate the flexibility of genSMR models and provide an illustrative example of the effectiveness of this combined sampling and analytical approach to produce precise and reliable population density estimates over large geographical areas.

## Materials and Methods

### Study area

**Figure 1:**
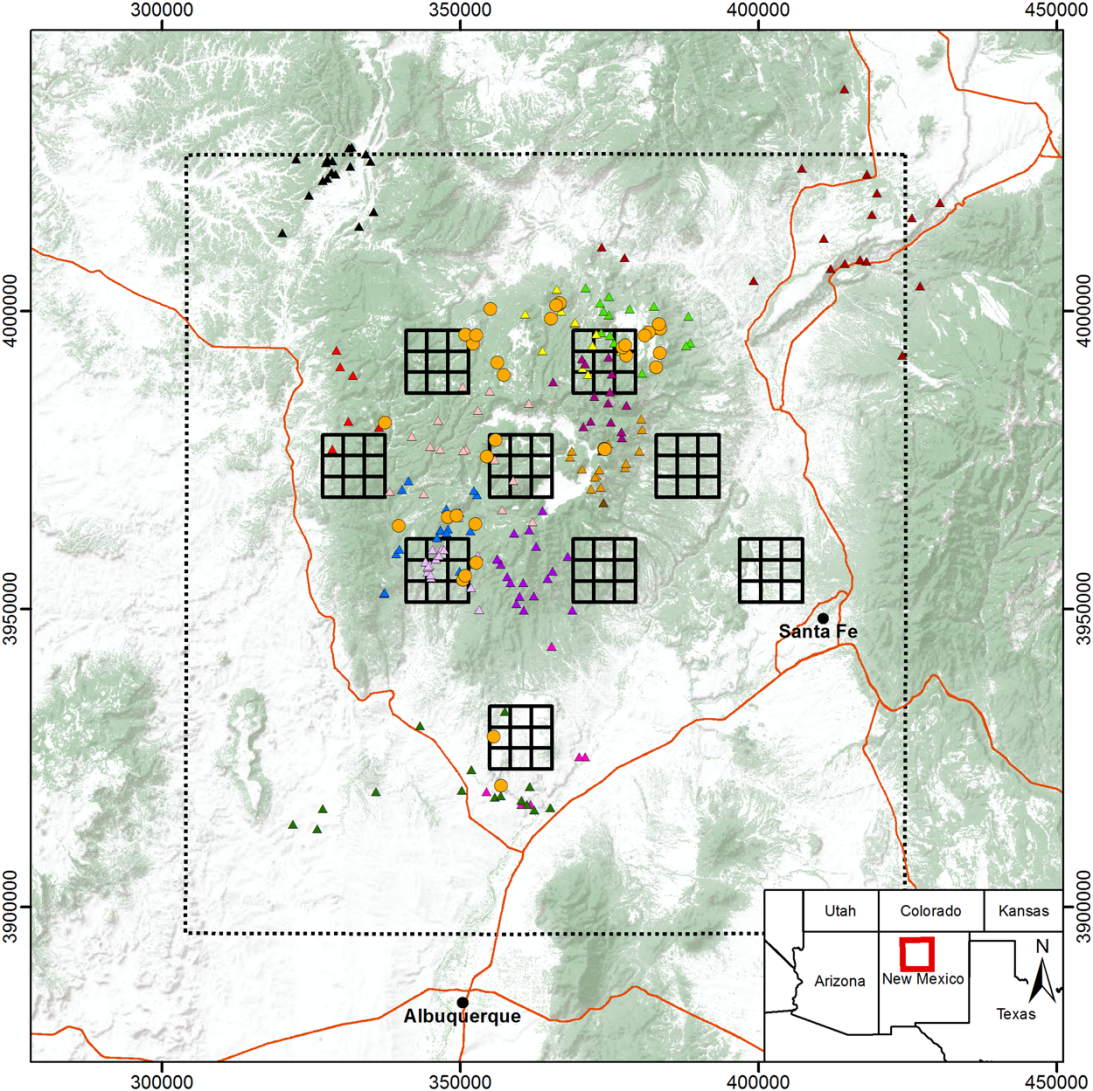
Study area in New Mexico, USA, where pumas were live-captured and marked with GPS collars, and camera-traps were deployed in a systematic cluster design for resighting of marked and unmarked pumas to estimate population density with generalized spatial mark-resight models. The spatial locations of live-traps (orange circles), camera-trap sampling cells (solid black outline squares), thinned telemetry locations collected during the resighting period (triangles with discrete colors corresponding to individual), and parameter estimation area (state space; dashed black line) are presented. Image created by S.M.M. with ESRI® ArcMap™ 10.4.1 software (http://desktop.arcgis.com/en/) under license (https://technology.ky.gov/gis/Pages/PostSecondarySiteLicense.aspx), with forest-shrub land cover data (green shaded areas) from the U.S. Government (https://www.mrlc.gov/data/nlcd-2011-land-cover-conus)^79^; topography data (background) from ESRI, U.S. Geological Survey, and National Oceanic and Atmospheric Administration (https://server.arcgisonline.com/ArcGIS/rest/services/World_Terrain_Base/MapServer); and major highways data (red lines) from New Mexico Department of Transportation (http://services.arcgis.com/hOpd7wfnKm16p9D9/arcgis/rest/services/NMDOT_Functional_Class/FeatureServer).

Our study occurred during 2017 in the Southern Rocky Mountains ecoregion in north-central New Mexico, USA (Fig. 1). The area was rugged, with steep mountains, deep canyons, and expansive mesas, and elevations ranging from 1,540 to 3,524 m a.s.l. The climate was semi-arid, with average annual rainfall ranging from 22.58 to 57.63 cm and average annual snowfall ranging from 18.03 to 305.31 cm, depending on elevation; average annual high temperatures ranged from 13.72 to 22.05 °C and average annual low temperatures ranged from − 4.17 to 3.00 °C, depending on elevation^46^. The majority of lands (63%) were under federal management by the U.S. Forest Service, National Park Service, or Bureau of Land Management; tribal lands (29%) and a combination of state government, local government, and privately owned lands (8%) accounted for the remainder of land area.

### Live-capture and marking

To apply artificial marks to a portion of individuals, we live-captured pumas throughout our study area using Aldrich spring-activated foothold cable restraints, foothold traps, and to a lesser extent, treeing with a team of trained hounds^47,48^. We chemically immobilized captured pumas using one of the following drug combinations^49^: (1) tiletamine and zolazepam (Telazol^®^; Zoetis Services LLC, Parsippany, USA) at a dosage of 5.0 mg/kg combined with 1.0 mg/kg of xylazine (AnaSed®, LLOYD Inc., Shenandoah, USA), the latter of which was antagonized using 0.12 mg/kg of yohimbine (ZooPharm, Windsor, USA); or (2) 2.0 mg/kg of ketamine combined with 0.07 mg/kg of medetomidine, the latter of which was antagonized using 0.30 mg/kg of atipamezole (ZooPharm). During immobilization, we monitored the respiratory rate, heart rate, and body temperature of each puma at five-minute intervals to ensure maintenance of bodily function. We outfitted captured pumas that were field-aged based on gum recession measurements^50^ as being ≥ two years-old (i.e., subadults and adults)^48^ with a uniquely numbered ear tag and an Iridium GPS collar (Advanced Telemetry Systems [Isanti, USA] or Vectronic Aerospace [Berlin, Germany]). We programmed collars to acquire location fixes every one to three hours (i.e., 8–24 fixes per calendar day) and we remotely downloaded location data every three to seven days. All pumas were released at the location where captured.

### Clustered camera-trap resighting

We created a survey design comprised of nine total clusters of 3 × 3 sampling cells in each cluster (Fig. 1). Cell spacing within a cluster was 3.5 × 3.5 km, or 12.25-km^2^ coverage per cell and 110.25-km^2^ coverage per cluster; this spacing corresponded to the recommended ≥two detectors within the smallest female home range size^43,45^ reported for pumas in New Mexico (30.10 km^2^)^51^. Clusters were staggered with 28-km longitudinal spacing and 36–45-km latitudinal spacing between the centers of clusters, or 4.5–7× the diameter of said smallest female home range size, assuming a bivariate normal distribution (i.e., circular home range)^19^. Prior to deploying camera-traps, we used simulation to evaluate the performance of this clustered survey design for estimating population density, given pessimistic parameter estimates and various numbers of sampling occasions^19,41,45^. For a simulated hypothetical population with low density (1.0 puma/100 km^2^), low baseline detection rate (λ_0_ = 0.05), and large spatial scale of the detection function (σ = 5.0 km)^20,25^, results from a fitted null spatial capture-recapture model indicated that surveying this design for 17 consecutive occasions would likely estimate population density with high precision and accuracy (CV = 0.18; RMSE = 0.19), negligible bias (+0.05, 95% CI = 0.00–0.09), and nominal coverage (0.97, 95% CI = 0.94–1.00; see Supplementary Table S1). These simulations assumed that all individuals had unambiguous identities, which deviates from the mark-resight framework, but the effectiveness of survey designs for spatial capture-recapture and SMR models are similar^19^.

We attempted to establish a single camera-trap within each sampling cell along canyon rims, ridges, saddles, drainages, trails, and other terrain features that could be likely travel routes for pumas; we did not place camera-traps on roads. Because of restricted property access, we were unable to establish camera-traps in some cells; thus, our final array was comprised of 68 total camera-traps (range: 3–9 camera-traps/cluster). Each camera-trap consisted of two cameras with passive infrared motion-activated sensors (Reconyx® HyperFire PC800; Holmen, USA), which we placed four to six m apart, facing each other, and mounted to trees or shrubs ~one m above the ground^52^. We set cameras to medium sensitivity with bursts of five photos per detection and 30-s delays between bursts. We placed ~1.0 mL of bobcat (*Lynx rufus*) gland-based or rub-eliciting scent lure on the ground in the center of each camera-trap. These lures provided no caloric reward, and felids do not have the extraordinary olfactory capabilities that canids and ursids do^53^; neither pumas, jaguars, nor leopards (*Panthera pardus*) have exhibited a behavioral response (i.e., trap-happy or trap-shy) to detection when bobcat lure was applied^54–56^. If a camera-trap is visited, however, bobcat lure can entice pumas to linger for a slightly extended period of time, thereby affording researchers the opportunity to identify the sex and marked status of an individual from photographs^24,57,58^.

We operated camera-traps for 17 consecutive seven-day occasions from July to November 2017, and we visited each camera-trap every 21–28 days to retrieve photographs, check battery levels, and reapply lure. We considered individual photographs of pumas that were acquired ≥one hr apart as unique detections^24,25^. We excluded dependent kittens, which are not reproductively mature, from the detection history to prevent inflation of density estimates^13,20^; therefore, our results represent subadult and adult pumas only. We first classified photographs by the mark status of each puma based on the presence or absence of a GPS-collar: (1) marked and identifiable, (2) marked but unidentifiable, (3) unmarked, or (4) unknown. We then identified marked pumas to the individual level based on a combination of ear tag, collar type, sex, and telemetry locations from GPS collars^26,37^. We did not attempt to assign individual identities to any non-collared pumas based on perceived natural marks, because of the inherent uncertainty that could bias density estimates^24^. We reclassified all pumas that we initially assigned unknown mark status as unmarked if photograph date and time did not align with telemetry location data for GPS-collared individuals. Similarly, we resolved all cases of marked but unidentifiable individuals by comparing telemetry locations with photograph date and time. We identified the sex of unmarked pumas when possible; for photographs from which puma sex was inconclusive, we assigned individuals unknown sex.

### Spatial mark-resight analysis

We estimated puma population density using the live-capture history (marking), the camera-trap detection history (resighting), and the telemetry locations from GPS-collared pumas. Because only two pumas were captured and marked via treeing with hounds, we did not explicitly model a separate hound capture process; however, we retained hound-captured pumas in our data as marked individuals that were exposed to both the marking and resighting processes, and they also provided telemetry data that informed their activity center locations and contributed to estimation of the detection function spatial scale parameter. To jointly use all of those sources of information and account for dependency among data types, we used a Bayesian genSMR model^37^ that specified a spatial capture-recapture density and activity center process model that was observed in three ways: (1) through the marking process in which all individual identities were known; (2) through the resighting process in which only the individual identities of marked pumas were known and unmarked identities could be partially known if sex was observed; and (3) through the telemetry process for the marked individuals with known identity. To reduce the uncertainty in probabilistically resolving the latent identities of unmarked individuals^34^, we used sex as a categorical identity covariate to exclude particular combinations of detections^39,40^; for example, an unmarked male detection could not be from the same individual as an unmarked female detection. This assumed that the sex of individual *i*, *sex*_*i*_ ~ Bernoulli(*p*^*sex*^), where *p*^*sex*^ is the probability that an individual is female, which must be estimated. Using this assumption, sex can be probabilistically resolved for detections of individuals whose sex was not identified from photographs^22^, and the individual identities of unmarked pumas can be probabilistically resolved using the algorithms developed by Chandler and Royle^34^, excluding identity matches between detections of different sexes. We also fit conSMR models, which ignore the marking process^26,34,36^, to permit comparisons with genSMR models. We accommodated all of the aforementioned features using MCMC algorithms that are maintained in the R statistical software package SPIM^59,60^.

We considered the following two process models for activity centers (***s***). First, we used a typical spatial capture-recapture point process model in which individual *i* had a single ***s***_*i*_ for the entirety of the study (marking and resighting combined), and all ***s***_*i*_ were uniformly distributed across space (***s***_*i*_ ~ Uniform(***S***) for *i* = 1, …, *N*, where ***S*** denotes the two-dimensional state space [parameter estimation area])^19^. To define the state space for genSMR models, we buffered the minimum and maximum longitude and latitude extents of the combined live-trap and camera-trap locations by 25 km, or ~3× the maximum estimated spatial scale of the detection function parameter that was pooled between marking and resighting processes (σ^*d*^)^19^, resulting in ***S***^*G*^ = 15,314 km^2^. In contrast, because conSMR models do not incorporate the marking process, the 25-km buffer was applied only to the camera-trap locations to define a state space for conSMR models of ***S***^*C*^ = 14,707 km^2^. Second, GPS-collar telemetry data indicated that the activity centers for four marked pumas may have spatially shifted large distances between the marking and resighting processes, and one marked puma died prior to the onset of resighting (see Results). Therefore, we also specified a spatial point process model for activity center transiency, which estimated the locations of individuals’ activity centers separately for each the marking and resighting processes^61,62^. This process model accommodated activity center relocations between marking and resighting, including if individuals relocated to fill the territorial vacancy that resulted from the death of one marked puma^63,64^. An individual’s activity centers were connected by a spatially constrained relocation event (described in detail below), which entailed that resighting activity centers must be spatially linked to the location where each marked puma was live-captured, thereby constituting an activity center model that was intermediate between conSMR and genSMR models^61,62^.

We defined data for the marking and resighting processes using the *M* and *R* superscripts, respectively. The previously mentioned two-step process model for genSMR models required us to specify two sets of activity centers, ${s}_{i}^{M}$ and ${s}_{i}^{R}$, for *i* = 1, …, *N*. We assumed spatial uniformity of activity centers for the marking process, ${s}_{i}^{M}$ ~ Uniform(***S***^*G*^). For the resighting process, we assumed ${s}_{i}^{R}$ ~ Bivariate Normal(${s}_{i}^{M}$, Σ)[(*x*_min_, *y*_min_), (*x*_max_, *y*_max_)], where Σ = σ^*t*^***I***, and σ^*t*^ is the spatial scale parameter for activity center transiency; the bivariate normal redistribution kernel was truncated by the extent of ***S***^*G*^ to prevent σ^*t*^ underestimation^62^. This model for redistribution (i.e., spatial shift) has been used in both open and closed population spatial capture-recapture models^62,65^, the latter of which allowed fully transient activity centers and was recently applied to conSMR models^61^. In contrast to those implementations, we only allowed one spatial redistribution of activity centers, because that was all that was necessary to accommodate the spatial dynamics that we observed, and fewer activity center shifts should maintain greater precision and better MCMC mixing, which is typically poor for spatially explicit models that accommodate transient activity centers^61,62^.

Conditional on the aforementioned process models, the population was observed via three processes. For the marking and resighting processes, observations were made at the *J*^*M*^ × 2 live-trap locations *X*^*M*^ and the *J*^*R*^ × 2 camera-trap locations *X*^*R*^, where *J*^*M*^ and *J*^*R*^ are the number of live-trap and camera-trap locations, respectively. We assumed a hazard half-normal detection function with binomial detections for the marking process, producing individual by live-trap detections summed across occasions, ${Y}_{ij}^{M}$ ~ Binomial(${p}_{ij}^{M}$, *K*^*M*^), where *K*^*M*^ is the number of marking occasions. For the resighting process, we assumed a Poisson detection function, producing individual by camera-trap counts that were summed across occasions; specifically, ${Y}_{ij}^{R}$ ~ Poisson(*K*^*R*^ × ${p}_{ij}^{R}$), where *K*^*R*^ is the number of resighting occasions. These observation models had σ^*d*^ and baseline detection rate parameters that varied by process (${\lambda }_{0}^{M}$ and ${\lambda }_{0}^{R}$). Telemetry locations from GPS collars could be recorded anywhere within the extent of ***S***. We used only the telemetry locations that were collected during the resighting period, which we thinned to one randomly selected location per survey occasion for each marked puma (i.e., one location/week). We applied this thinning to decrease temporal dependence among telemetry locations for each puma, because temporal dependence could cause underestimation of the variance of σ^*d*^ and σ^*t*^, activity centers, and population density^26,36,37^. Telemetry locations informed the estimation of σ^*d*^ and ***s***_*i*_, or σ^*d*^, ${s}_{i}^{R}$, and σ^*t*^ for models that included activity center transiency.

We accounted for unequal live-trap and camera-trap operation (effort) across time, and also a puma that died prior to initiation of resighting, using individual by trap exposure matrices. These matrices are similar to a trap operation file^19^, except that the exposure of each puma to each trap and trap type could differ; this allowed for known entries and exits into and out of the population, but did not account for unknown violations of the population closure assumption^37,39^. For the marking process, the *A* × *J*^*M*^ exposure matrix *E*^*M*^ contained the number of occasions that individual *i* was exposed to detection at a live-trap *j*, where *A* indicates the level of data augmentation^66^. For the resighting process, the *A* × *J*^*R*^ exposure matrix *E*^*R*^ contained the number of occasions that individual *i* was exposed to detection at camera-trap *j*. These exposure matrices were substituted into the binomial and Poisson observation models for *K*^*M*^ and *K*^*R*^, respectively. To correctly allocate latent identity samples for two pumas that were live-captured and marked during the resighting period and one marked puma that died prior to resighting, we used an *n*^*M*^ × *K*^*M*^ matrix *m*, where *n*^*M*^ is the number of marked pumas, to denote the marked status of each GPS-collared puma during each resighting occasion (0 = unmarked, 1 = marked, and 2 = dead)^37^. Thus, if a puma was unmarked on occasion *k*, it could be allocated latent identity unmarked detections. If a puma was marked on occasion *k*, it could be allocated latent identity marked detections. If a puma was dead on occasion *k*, it could not be allocated any latent identity detections.

Several process and observation models were described, so we detail below exactly which combinations we fit. Our model specifications were designed to test the relative importance of four items: (1) telemetry data from marked pumas, (2) sex as a categorical identity covariate for unmarked pumas, (3) activity center transiency for marked pumas between the marking and resighting processes, and (4) conSMR versus genSMR models. The influence of telemetry data was of particular interest, because the activity centers for four marked pumas likely relocated between marking and resighting, and we also had limited prior home range size data to inform camera-trap and cluster spacing. Therefore, we fit two genSMR models that included sex identity constraints for the resighting process, but differed as to whether telemetry data were incorporated or not (models 1 and 2). We extended models 1 and 2 to accommodate activity center transiency between the marking and resighting processes for marked pumas (models 3 and 4). Because models 3 and 4 best described the observed spatial dynamics of pumas during our study, we tested the importance of sex identity constraints by fitting these models without sex identity constraints (models 5 and 6). To test the importance of using genSMR over conSMR models, we fit models 1 and 2 excluding the marking process (models 7 and 8). Finally, to investigate if sex-specific detection function parameters were necessary to estimate puma density and the sex ratio, we fit a version of model 1 that included sex-specific detection function parameters (model 9).

We ran each genSMR model for 5 × 10^5^ iterations, thinned by 75 iterations, and we discarded the first 5 × 10^3^ iterations as burn-in. The large number of iterations was more than required for the models that excluded activity center transiency, but for models that included activity center transiency, σ^*t*^ mixed poorly and required many iterations to accurately characterize this posterior distribution. In contrast, we ran each conSMR model for 4 × 10^4^ iterations and discarded the first 5 × 10^3^ iterations as burn-in. We used data augmentation to augment the sample of marked pumas with up to *A* = 250, 325–375, and 600 hypothetical individuals that had all-zero detection histories for conSMR models, genSMR models that included telemetry data, and genSMR models that excluded telemetry data, respectively^26,36,37,66^. We used the posterior modes for parameter point estimates, and we used the 95% highest posterior density intervals (HPDI) for interval estimates. We assessed precision of density estimates using the widths of 95% HPDIs and the posterior coefficients of variation (CV), or the posterior standard deviation divided by the posterior mode.

#### Ethics statement

Experimental protocols were approved by New Mexico Department of Game & Fish (per NMAC 19.35.6), Pueblo of Santa Ana Tribal Council, and a U.S. National Park Service Institutional Animal Care and Use Committee (IMR-VALL-Cain-LargeMammals-2015.A2). Data collection methods were carried out in accordance with standardized guidelines for humane wild mammal handling and welfare^67^, scientific research permits (VALL-2017-SCI-0002 and VALL-2017-SCI-0049), and with explicit permission from relevant authorities.

## Results

### Marking and resighting

We deployed 30 live-traps, each for an average of 22 days (range: 2–64 days). We live-captured and marked 15 pumas (12 males:3 females); one marked female died of starvation prior to initiation of camera-trapping. We used a total of 190 telemetry locations (*n*_males_ = 156; *n*_females_ = 34) collected from GPS collars during the resighting period (mean = 14 locations/puma; range = 3–17). We acquired 68 unique detections of subadult and adult pumas at 31 camera-traps (46% of traps); the average number of detections per occasion was four (range: 1–7). Twenty (29%) camera-trap detections were of eight marked pumas (6 males:2 females); 17 spatial recaptures of marked pumas were obtained during the marking and resighting processes combined (*n*_males_ = 15; *n*_females_ = 2). Among the 48 detections of unmarked pumas, sex was definitively identified for 25 detections (52%; 10 male:15 female).

### Population density and abundance

Puma population density point estimates ranged from 0.66 to 1.65 pumas/100 km^2^, with the lowest estimates produced by conSMR models and the highest estimates produced by genSMR models that excluded telemetry data (Table 1). Integrating telemetry data approximately doubled σ^*d*^ estimates and decreased estimates of puma density in the genSMR models, whereas estimated puma density from conSMR models were similar regardless of whether telemetry data were used or not (0.66 versus 0.70 puma/100 km^2^, respectively). The estimated number of unmarked pumas that were detected during resighting (*n*^*UM*^) was between 18 and 26 individuals, with the smallest estimates from conSMR models (18–20 pumas) and the genSMR models that excluded telemetry data (20–22 pumas). The genSMR model that included telemetry data, activity center transiency, and sex as a partially identifying categorical covariate (model 3), which best explained the observed spatial dynamics of pumas during our study, estimated population density to be 0.84 puma/100 km^2^ (95% HPDI: 0.50–1.28) with a CV of 0.24. This corresponded to an estimated population size of 129 pumas (95% HPDI: 74–193) across the 15,314 km^2^ estimation area, of which an estimated 26 unmarked pumas (95% HPDI: 18–32) were detected by camera-traps. Given those point estimates, 11.63% of pumas were marked and 22.81% of unmarked pumas were detected by camera-traps, indicating that we acquired spatial detection information for a combined 34.44% of pumas within ***S***^*G*^.

**Table 1:** Parameter estimates from generalized (Gen) and conventional (Con) spatial mark-resight models.

Model	Type	Specifications	${\lambda }_{0}^{M}$	${\lambda }_{0}^{R}$	σ^*d*^	σ^*t*^	*n* ^UM^	*D* (95% HPDI)	Width	CV	*N* (95% HPDI)
1	Gen	Sex + Tel	0.004	0.016	7.54	—	25	0.94 (0.59–1.48)	0.89	0.25	144 (91–227)
2	Gen	Sex	0.016	0.061	2.85	—	22	1.54 (0.96–2.75)	1.79	0.31	236 (147–421)
3	Gen	Sex + Tel + Trans	0.007	0.019	6.51	17.40	26	0.84 (0.50–1.28)	0.78	0.24	129 (74–193)
4	Gen	Sex + Trans	0.018	0.064	2.89	0.35	22	1.57 (0.93–2.65)	1.72	0.29	240 (142–406)
5	Gen	Tel + Trans	0.008	0.020	6.54	17.02	26	0.84 (0.54–1.34)	0.81	0.26	129 (82–206)
6	Gen	Trans	0.021	0.068	2.63	2.71	20	1.65 (0.95–2.72)	1.77	0.29	252 (145–417)
7	Con	Sex + Tel	—	0.025	6.64	—	20	0.66 (0.37–1.03)	0.66	0.26	97 (55–151)
8	Con	Sex	—	0.082	3.62	—	18	0.70 (0.33–1.27)	0.94	0.37	102 (49–187)
9	Gen-SS	Males + Tel	0.005	0.015	8.10	—	24	0.95 (0.59–1.43)	0.84	0.24	145 (90–219)
		Females + Tel	0.005	0.042	4.22	—					

Models with and without a categorical identity constraint for puma sex (Sex), telemetry data from GPS collars (Tel), activity center transiency between marking and resighting processes (Trans), and sex-specific detection functions (SS) were considered. Baseline detection rates for the marking (${\lambda }_{0}^{M}$) and resighting (${\lambda }_{0}^{R}$) processes, spatial scale of the detection function (σ^*d*^; km), spatial scale of activity center transiency (σ^*t*^; km), the number of unmarked pumas detected duri*n*g resighting (*n*^UM^), population density (*D* = puma/100 km^2^), and population size (*N*) were estimated. The 95% highest posterior density intervals (HPDI) are presented for *D* and *N*, as well as 95% HPDI width and coefficient of variation (CV = S*D*/*D*) for *D*. See Supplementary Table S2 for further details, including 95% HPDIs for all parameter estimates.

### Density estimate precision

Modeling sex as a partially identifying categorical covariate for the detections of unmarked pumas improved precision of estimated density by 8%, reducing CV from 0.26 to 0.24 (model 5 versus model 3). Allowing activity center transiency for marked pumas between the marking and resighting processes improved precision of estimated puma density by 4% (based on CV), despite introducing more uncertainty into the process model via more complex model structure. Integrating telemetry data from GPS collars on marked pumas improved precision of estimated density by 17%, reducing CV from 0.29 to 0.24 (model 4 versus model 3); although, determining how much of the CV reduction resulted from a lower point estimate instead of a decrease in variance is difficult to disentangle.

### Spatial scale of detection and activity center transiency

**Figure 2:**
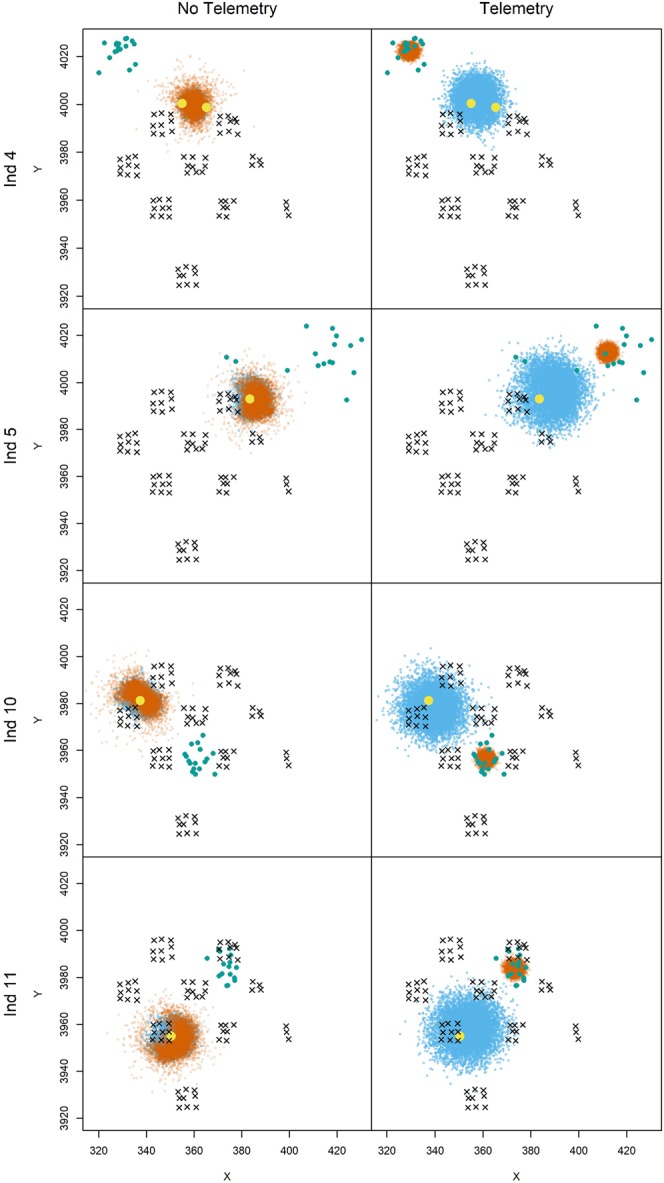
Estimated activity center locations for four marked pumas from generalized spatial mark-resight models that accommodated activity center transiency between marking and resighting processes, and excluded or included telemetry location data from GPS collars. The estimated posterior densities of individual activity centers for the marking and resighting processes are denoted by blue and orange, respectively. The spatial locations where each puma was live-captured, the locations of camera-traps, and thinned telemetry locations from the resighting period are denoted by yellow circles, black × , and green circles, respectively. Image created by B.C.A. with the R statistical software^60^.

Estimates of σ^*d*^ from models that incorporated telemetry data ranged from 6.51 to 7.54 km, whereas estimates from models that excluded telemetry data ranged from 2.63 to 3.62 km. The smallest estimated σ^*d*^ was from the genSMR model that only included activity center transiency (model 6), whereas the largest σ^*d*^ was from the genSMR model that excluded activity center transiency but incorporated sex identity constraints and telemetry data (model 1). Estimated σ^*t*^ was 17.40 and 17.02 km from genSMR models that included both activity center transiency and telemetry data (models 3 and 5, respectively), but was just 0.35 and 2.71 km from genSMR models that excluded telemetry data (models 4 and 6, respectively). In models 4 and 6, σ^*t*^ was either not identifiable or was barely identifiable, so these considerably lower estimates are likely unreliable. Importantly, telemetry data from the GPS-collared pumas were critical to estimating σ^*t*^, because the four individuals whose activity centers relocated between the marking and resighting processes were never detected by the camera-traps (Fig. 2).

### Sex ratio

The genSMR model that included sex-specific detection functions (model 9) produced a similar population density estimate as the comparable genSMR model that had a pooled detection function (model 1). The estimated female and male σ^*d*^ from model 9 was 4.22 km (95% HPDI: 3.65–5.10) and 8.10 km (95% HPDI: 7.57–8.61), respectively, compared to the pooled estimate from model 1 of 7.54 km (95% HPDI: 7.06–8.12). The probability that a puma was female was 0.33 (95% HPDI: 0.16–0.49) and 0.34 (95% HPDI: 0.19–0.52) from models 3 and 9, respectively, which supports that sex-specificity of detection function parameters was unnecessary for accurately estimating the population sex ratio. The fact that the density and sex ratio estimates were nearly identical between models with and without sex-specificity suggests close to perfect compensation between ${\lambda }_{0}^{R}$ and σ^*d*^ on the total exposure to detection^68^. We note that with just two spatial recaptures for marked females, our female density and sex ratio estimates are largely dependent on how representative the telemetry data (i.e., movements) for the two marked females were of the entire female cohort within ***S***^*G*^.

## Discussion

Previous puma mark-resight studies in the spatially explicit framework used conSMR models to estimate population density^25–27^. If individual animals are live-captured to apply artificial marks, and this process occurs across the same area in which resighting will occur, marked individuals will on average likely reside closer to the resighting array than unmarked individuals^37^. Modeling the marking process via genSMR models accounts for these spatial patterns in activity centers, but conSMR models exclude the marking process and consequently may produce negatively biased density estimates^37,39^. Indeed, our puma density estimates from conSMR models were ~17% lower than density estimated by our best genSMR model (model 3), chosen because of its most accurate characterization of the observed puma spatial dynamics (e.g., activity center transiency [through telemetry data] and spatial information about sex of unmarked pumas). Thus, our results support that genSMR models are preferable to conSMR models when the marking process involves live-capture and the marking and resighting arrays spatially overlap; particularly if researchers cannot assume that marked animals are uniformly distributed across the landscape, or the spatial distribution of marked animals is unknown and cannot be correctly specified.

Integrating telemetry data from GPS collars on marked pumas substantially improved parameter estimate precision and was critical for accurately estimating population density. First, the telemetry data allowed us to definitively determine individual identities from photograph detections. This was arguably more reliable than attempting to assign identities *ad hoc* based on researcher-perceived natural marks for a species that generally does not have unambiguous, individually unique physical features^24–27^. Although researchers may be tempted to treat all pumas detected by camera-traps as unmarked and apply the ‘unmarked’ spatial capture-recapture model^34^ to estimate population density, the large home ranges and generally low detection rates of pumas, regardless of sampling method, will likely result in biased, imprecise, and unreliable density estimates from this model^39,40^. Applying artificial marks to even a small portion of a population and using SMR models can greatly improve estimation of detection function parameters and population density^26,34,36,37,39^.

Telemetry data also facilitated accurate estimation of σ^*d*^, which our results suggest was substantially underestimated by the models that relied solely on camera-trap detection data (models 2, 4, and 6). To establish our clustered camera-trap design, we based simulations on parameter estimates from previously published spatially explicit puma density studies. Based on the σ that we used in simulations (5.0 km), we presumed that our camera-trap and cluster spacing were 0.70σ and 5.60–7.20σ, respectively; however, based on the σ^*d*^ estimated by our best model (model 3), camera-trap and cluster spacing turned out to be 30% smaller (0.54σ^*d*^ and 4.30–5.53σ^*d*^, respectively). If home ranges are large and detection rates are low (λ_0_ < 0.10), detector spacing as small as 0.5σ may be too close to accurately characterize the true scale of animal movement within a single cluster^43,45^. Estimated ${\lambda }_{0}^{R}$ was <0.10 among all of our considered models, and each of the nine clusters of camera-traps was considerably smaller than the average puma home range size derived from estimated σ^*d*^, assuming a bivariate normal distribution^19^ (110.25-km^2^ cluster size versus 799.23-km^2^ home range size, based on model 3). Consequently, the full extent of individual puma space use likely could not be captured within a single cluster^45^, which resulted in underestimation of σ^*d*^ and overestimation of puma density by the models that excluded telemetry data. Employing a wider camera-trap spacing of 1–2σ^*d*^ (6.51–13.02 km) within each cluster likely would have resulted in detections via the camera-traps alone that more accurately reflected the larger than expected puma space use^45^. Although our spacing between clusters was well within the movement capabilities of pumas in the study area (based on estimated σ^*d*^), a wider camera-trap spacing within clusters would also decrease the distance between clusters, which might have the added benefit of increasing the number of spatial recaptures^43,45^.

An alternative but unlikely explanation for the smaller σ^*d*^ and higher puma density estimates from models that excluded telemetry data could be that the marked pumas were not a random sample of the population, but were instead representative of a cohort of pumas that had larger than average home ranges^36^. Subadult male pumas are generally transient and typically have the largest home ranges among all sex-specific cohorts of puma populations^69^. We live-captured and marked both subadults and adults and both males and females, however, and although just 20% of our marked pumas were females, genSMR model results suggested that only 33–34% of the population was female. Furthermore, the point and interval estimates of puma density from the genSMR model with sex-specific detection function parameters (model 9) were nearly identical to the analogous model with detection function parameters pooled between sexes (model 1). This strongly supports that a sex imbalance among marked individuals was not a source of incongruous σ^*d*^ estimates between models that included and excluded telemetry data, thereby indicating that density estimates from the genSMR models that integrated telemetry data more accurately reflected puma space use during our study.

A third reason supporting the importance of telemetry data, and a primary reason why the transient activity center model improved density estimation, was to accurately estimate activity center locations for the pumas who relocated considerable distances between the marking and resighting processes. Efford and Hunter^35^ raised concerns about the potential for such activity center transiency between observation processes to influence SMR model parameter estimates, but those authors had no independent data to test for this. In contrast, the telemetry data that we had from marked pumas allowed us to document and model large activity center relocations between processes. Because the four marked pumas who relocated were not detected by camera-traps, the resighting data provided little information about whether or not those individuals’ activity center locations moved, and if so, how far. Although two pumas (individuals 10 and 11) moved to areas of the camera-trap array where they likely had similar detectability as the locations at which they were live-captured and marked, two other pumas (individuals 4 and 5) moved to areas where they were effectively undetectable by all camera-traps (Fig. 2). In model 1, which did not accommodate activity center transiency, the distances between live-capture locations and the estimated activity center locations, which were primarily informed by the telemetry data, were larger than reality. This inflated the σ^*d*^ estimate (7.54 versus 6.51 km from models 1 and 3, respectively), which in turn decreased the ${\lambda }_{0}^{R}$ and ${\lambda }_{0}^{M}$ estimates. These differences in detection function parameters corresponded to a ~12% difference in puma density point estimates (0.94 versus 0.84 puma/100 km^2^), suggesting that accommodating activity center transiency may be important for reliably estimating population density in SMR studies. Additionally, σ^*t*^ was substantially underestimated without the telemetry data, because all four major movements were not discernable from the camera-trap data; this caused poor estimation of those pumas’ activity center locations and introduced bias into detection function and density parameter estimates. Thus, having considerable telemetry data likely will lead to a more robust application of SMR models, informing if activity center transiency needs to be accommodated in the model structure to improve parameter estimation.

Fully transient activity centers have been considered in conSMR models^61^, but our study is the first application of a single activity center transition that was used to explain observed animal movement dynamics. The base genSMR model provides an adequate description of the distribution of marked and unmarked individuals if they do not relocate between the marking and resighting processes; if individuals randomly relocate between processes, which is unlikely, the spatial uniformity activity center model may be appropriate. Accommodating activity center transiency as we did results in an intermediate activity center model in which individuals are not at exactly the same spatial location between processes and the similarity of locations is determined by the σ^*t*^ parameter. However, if individual animals exhibit multiple substantial movements during observation processes, an activity center model that accommodates fully transient activity centers might be more appropriate^61,62^. Nevertheless, distinguishing between a process model with stationary activity centers and a large σ^*d*^ value and a model with transient activity centers and a small σ^*d*^ value will be difficult without considerable telemetry data, given the sparsity of typical capture-recapture and mark-resight detection data.

Despite the relatively small improvement in density estimate precision from using sex as a categorical identity covariate compared to the substantial improvement from incorporating telemetry data, using categorical identity covariate data that is available from camera-trap detections has considerable promise. The 8% precision improvement that we observed by using sex of unmarked pumas comes from data that has not been used in SMR models to date, but ecologists and managers should be interested in extracting as much precision out of detection data as possible. Additionally, sex was a single categorical identity covariate that we confirmed for only approximately half of the detections of unmarked pumas. Other populations of pumas or other wildlife species may provide more categorical identity covariate information from photographs; for example, the natural marks used by previous studies to attempt to assign individual identities for estimating population density^24,25,27,61,70^ could instead be treated as categorical identity covariates, allowing for the possibility that more than one individual in a population has a similar physical feature. This would obviate the requirement that potentially erroneous individual identities are assigned, but it may also reduce the precision of density estimates, perhaps appropriately, depending on the accuracy of categorical identities assigned by observers.

We acknowledge that using GPS collars as the primary mark can be expensive, but our results indicate that the realized and potential benefits of marking a portion of a population with GPS collars outweigh the costs. Clearly, integrating telemetry data in spatially explicit analyses can substantially improve estimation of the spatial scale parameter, activity center locations, and population density, as also noted by previous studies^26,36,37,39^. Furthermore, by marking a portion of animals with GPS collars, which are typically functional for multiple years, additional demographic and ecological information that are important to conservation and management can be obtained, effectively constituting SMR as a population ecology research approach. This includes data on survival and cause-specific mortality, home range size, and resource selection^71,72^, as well as seasonal and annual variation in population density if camera-traps are active across seasons and years, respectively. Additionally, if population genetics are of interest, genetic samples can be collected when animals are captured for marking. If study budgets are limited, a cheaper alternative may be to mark some animals with GPS collars and others with only ear tags or non-GPS collars that have visually unique numbers or patterns that can be identified from photographs. For example, Whittington *et al*.^37^ GPS-collared some individuals, only ear-tagged others, and used camera-traps and genSMR models to precisely estimate brown bear (*Ursus arctos*) population density.

Pumas occupy tens to hundreds of thousands of square kilometers within most jurisdictions across their extant range^1,69,73^. In general, precision and accuracy of spatially explicit population density estimates for wide-ranging large carnivores improve with increasing study area size^44,45,74^. By deploying camera-traps in a systematic cluster design with gaps between clusters where no cameras existed, we were able to use a small number of camera-traps to estimate puma density for a 15,317-km^2^ area. This area was five-fold larger than the average spatial extent among all previous puma density studies that also used spatially explicit models (mean = 2,849 km^2^; range: 215–8,800 km^2^), and our density estimates were among the most precise estimates that have been produced for pumas to date (CV_[genSMR]_ = 0.24–0.31; Table 2). Therefore, clustered camera-trapping in an SMR framework can facilitate efficient and reliable estimation of puma population density at the broad regional scales that conservation and management typically occur. For example, endangered Florida panthers (*P. c. coryi*) reside within a ~16,000-km^2^ area that encompasses multiple patches of suitable habitat^75^, and a portion of panthers are annually captured and collared^26,76^. Applying clustered camera-trapping across that entire area and using genSMR models to analyze detection data could result in the first range-wide spatially explicit estimates of Florida panther population density and abundance, with little additional effort compared to other available puma sampling approaches in the spatially explicit framework. Our sampling and analytical combination is likely also applicable to other terrestrial mammals that similarly lack individually unique natural markings. For instance, obtaining reliable population density and abundance estimates for imperiled Mexican gray wolves (*Canis lupus baileyi*) and red wolves (*C. rufus*) is important to their recovery, and individual wolves in those populations are routinely monitored via radiocollars that could serve as effective marks. Nevertheless, we agree with other studies that suggested researchers should use simulation to develop study area- and species-specific survey designs prior to deploying camera-traps^43,45,74^. Having home range size data beforehand to inform camera-trap and cluster spacing would be ideal^45^, but if such data are unavailable, our results support that marking a portion of animals with GPS collars and integrating their telemetry location data in spatially explicit models can serve as insurance if detector spacing turns out to be insufficient^36^.

**Table 2:** Study locations, sampling methods, model types, and parameter estimation areas (km^2^) for studies that used spatial capture-recapture (SCR), conventional spatial mark-resight (conSMR), or generalized spatial mark-resight (genSMR) models to estimate puma population density (puma/100 km^2^), ordered by sampling methods and model types.

Study	Location	Methods	Models	Area	Densities	Widths	CVs
This study	New Mexico, USA	CC + TL	genSMR	15,314	0.84–1.65	0.8–1.8	0.24–0.31
Sollmann *et al*.^26^	Florida, USA	RC + TL	conSMR	1,719	1.46–1.51	1.9–2.2	0.33–0.38
Rich *et al*.^25^	Belize, Bolivia, Argentina	RC	conSMR	4,329*	0.30–6.50	0.5–8.1	0.26–0.38
Zanón-Martinez *et al*.^27^	Argentina	RC	conSMR	1,179*	1.38–4.90	3.3–5.9	0.31–0.66
Quiroga *et al*.^77^	Argentina	RC	SCR	1,882*	0.08–1.26	0.2–1.0	—
Noss *et al*.^78^	Bolivia	RC	SCR	215*	0.36–7.99	0.7–9.9	0.20–0.85
Alexander and Gese^24^	Wyoming, USA	RC	SCR	1,287	0.39–4.04^†^	0.6–9.9	—
Proffitt *et al*.^21^	Montana, USA	BD + SB + DR	SCR	5,912	3.20–5.60	2.9–14.0	—
Russell *et al*.^22^	Montana, USA	BD + SB	SCR	8,800	3.70–6.70	1.5–7.9	0.24–0.46
Beausoleil *et al*.^20^	Washington, USA	BD	SCR	7,939	1.90–2.40	3.2–3.9	—
Davidson *et al*.^23^	Oregon, USA	SD	SCR	1,225	2.31–5.50	1.2–5.8	—

Methods included biopsy darting (BD), snow-backtracking (SB), scat detection dogs (SD), regular camera-trapping (RC), clustered camera-trapping (CC), dead recoveries (DR), and telemetry locations from GPS collars (TL). Coefficient of variation (CV), standard errors, or standard deviations were not reported by multiple studies (—), so we also present 95% interval widths for comparing precision of density estimates. Densities are presented as the ranges of point estimates. *Average among multiple study areas; ^†^excludes one density estimate for which variance of the corresponding spatial scale parameter (σ) was inestimable.

Our study provides the first spatially explicit population density estimates for pumas in the semi-arid to arid southwestern United States, where hot summer temperatures, high ultraviolet radiation, and generally limited winter snow cover may impede effectiveness of, or preclude, scat detection dog and biopsy dart sampling of pumas. Regardless of model specification, all of our puma density estimates were within the range of reported spatially explicit estimates for the species, but density estimated by our best model (0.84 puma/100 km^2^) was towards the lower bound of that range (Table 2). Estimates acquired using the biopsy dart and scat detection dog methods may not be directly comparable to our estimates, however, because estimates from those techniques might be inflated as a result of including dependent juveniles in the detection histories^20,23^, whereas our estimates pertain solely to independent pumas. Nonetheless, the majority of our study area was characterized as high quality puma habitat relative to elsewhere in the Southwest^73^; thus, our estimates suggest that the Southwest might commonly support pumas at lower densities than ecosystems in the Northwest and Northern Rockies regions^20–24,51^. Additional research is needed to evaluate the influence that legal harvest of pumas and prey availability and distribution may have on seasonal and annual variation of puma population density in our study area and across the Southwest in general.

## Supplementary information

Supplementary Information

## Data Availability

All data generated for analysis and all R code of MCMC algorithms for reproducing the analysis are available from the PANGAEA^®^ digital repository, 10.1594/PANGAEA.897113. Data were made available under provisions of the State of New Mexico Inspection of Public Records Act (1978 NMSA 14.2).
