# Supplementary material for: Improving estimation of puma (Puma concolor) population density: clustered camera-trapping, telemetry data, and generalized spatial mark-resight models
Source: Sci Rep. 2019 Mar 14;9:4590. doi: 10.1038/s41598-019-40926-7 (PMC6418282; doi:10.1038/s41598-019-40926-7)
Supplement: Supplementary file 1 — Supplementary Information [file 41598_2019_40926_MOESM1_ESM.pdf]

Improving estimation of puma (*Puma concolor*) population density: clustered camera-trapping, telemetry data, and generalized spatial mark-resight models

Sean M. Murphy[1,5,*], David T. Wilckens[1], Ben C. Augustine[2], Mark A. Peyton[3] & Glenn C. Harper[4]

[1]Wildlife Management Division, New Mexico Department of Game & Fish, Santa Fe, 87507, USA
[2]Atkinson Center for a Sustainable Future, Department of Natural Resources, Cornell University, Ithaca, 14853, USA
[3]Valles Caldera National Preserve, U.S. National Park Service, Jemez Springs, 87025, USA
[4]Department of Natural Resources, Pueblo of Santa Ana, Santa Ana Pueblo, 87004, USA
[5]*Present affiliation*: Department of Forestry and Natural Resources, University of Kentucky, Lexington, 40546, USA

[*]smmurp2@uky.edu

ABSTRACT

This file contains the supplementary information cited in the main text. All data and R code for reproducing the analysis are available from the PANGAEA® digital repository, https://doi.pangaea.de/10.1594/PANGAEA.897113.

Supplementary Tables

Table S1. Results from simulations that evaluated the performance of our 3 × 3 clustered survey design for estimating density of a hypothetical puma population (D = 1.0 puma/100 km^2) with a low baseline detection rate (λ_0 = 0.05) and large spatial scale of the detection function (σ = 5.0 km). Said parameter estimates were sourced from previous puma spatial capture-recapture and spatial mark-resight studies[1,2]. We created a state space by buffering the centroids of sampling cells (hypothetical traps) by 3σ = 15.0 km, which resulted in an 11,437-km^2 rectangular parameter estimation area with 3.0-km mask point spacing (0.6σ)[3,4]. Using the R package secrdesign[4], we conducted 100 replicate MCMC simulations for each 8, 12, and 17 consecutive survey occasions (i.e., 300 total replicates), with traps modeled as proximity 'count' detectors (Poisson detection), and then fit null spatial capture-recapture models with a hazard half-normal detection function via maximum likelihood in the R package secr[5]. We discarded replicates that produced rogue estimates (i.e., were outside of the 95% lower and upper quantiles[4]; n = 10 replicates, or 3.3% of all replicates), averaged the remaining replicates for each occasion-specific scenario, and evaluated performance of the clustered design based on relative bias, coefficient of variation (CV = SE/D), root mean square error (RMSE), and 95% confidence interval coverage of density.

	**8 occasions**	**12 occasions**	**17 occasions**
**Metric**	**Estimate (95% CI)**	**Estimate (95% CI)**	**Estimate (95% CI)**
Coverage	0.92 (0.86–0.97)	0.96 (0.93–0.99)	0.97 (0.94–1.00)
Bias	0.12 (0.06–0.18)	0.10 (0.04–0.15)	0.05 (0.00–0.09)
CV	0.30	0.23	0.18
RMSE	0.32	0.30	0.19

Table S2. Parameter estimates, standard deviations (SD), and 95% highest posterior density intervals (lower and upper bounds) from generalized (Gen) and conventional (Con) spatial mark-resight models. Models that included (+) or excluded (–) sex as a partially identifying categorical covariate (Sex), telemetry data from marked pumas' GPS collars (Tel), and activity center transiency (Trans) were considered. Model 9 had sex-specific detection function parameters (SS), whereas models 1–8 had detection function parameters pooled between sexes. Baseline detection rates (λ_0) for the marking (λ_0^M) and resighting (λ_0^R) processes, pooled spatial scale of the detection function (σ^d; km), spatial scale of activity center transiency (σ^t; km), number of unmarked pumas detected (n^{UM}), population density (D = puma/100 km^2), and population size (N) were estimated.

Model	Type	Sex	Tel	Trans	Parameter	Estimate	SD	Lower	Upper
1	Gen	+	+	–	λ_0^M	0.004	0.002	0.002	0.008
					λ_0^R	0.016	0.004	0.009	0.025
					σ^d	7.537	0.271	7.059	8.119
					N	145	35.888	91	227
					n^{UM}	25	3.728	20	34
					D	0.944	0.234	0.594	1.482
2	Gen	+	–	–	λ_0^M	0.016	0.009	0.006	0.04
					λ_0^R	0.061	0.020	0.032	0.107
					σ^d	2.853	0.372	2.288	3.701
					N	236	72.290	147	421
					n^{UM}	22	3.097	16	28
					D	1.543	0.472	0.960	2.749
3	Gen	+	+	+	λ_0^M	0.007	0.004	0.003	0.018
					λ_0^R	0.019	0.005	0.013	0.033
					σ^d	6.514	0.236	6.177	7.099
					σ^t	17.404	4.610	11.261	27.811
					N	129	31.32	74	193
					n^{UM}	26	3.588	18	32
					D	0.842	0.204	0.503	1.28
4	Gen	+	–	+	λ_0^M	0.018	0.012	0.007	0.047
					λ_0^R	0.064	0.021	0.034	0.111
					σ^d	2.890	0.378	2.184	3.645
					σ^t	0.346	1.767	0.297	5.626
					N	240	69.731	142	406
					n^{UM}	22	3.034	16	27
					D	1.568	0.455	0.927	2.651
5	Gen	–	+	+	λ_0^M	0.008	0.004	0.003	0.017
					λ_0^R	0.020	0.005	0.012	0.032
					σ^d	6.544	0.239	6.195	7.119
					σ^t	17.025	5.843	10.868	27.722
					N	129	33.318	82	206
					n^{UM}	26	3.785	19	33
					D	0.842	0.218	0.535	1.345

Table S2 continued									
**Model**	**Type**	**Sex**	**Tel**	**Trans**	**Parameter**	**Estimate**	**SD**	**Lower**	**Upper**
					λ_0^M	0.021	0.015	0.007	0.057
					λ_0^R	0.068	0.024	0.035	0.126
					σ^d	2.627	0.382	2.087	3.520
6	Gen	–	–	+	σ^t	2.712	1.639	0.634	6.027
					N	252	73.085	145	417
					n^{UM}	20	3.369	14	27
					D	1.648	0.477	0.947	2.723
					λ_0	0.025	0.005	0.015	0.037
					σ^d	6.641	0.242	6.205	7.148
7	Con	+	+	–	N	97	25.344	55	151
					n^{UM}	20	3.581	16	29
					D	0.659	0.172	0.374	1.027
					λ_0	0.082	0.025	0.033	0.128
					σ^d	3.619	0.803	2.505	5.464
8	Con	+	–	–	N	102	37.510	49	187
					n^{UM}	18	2.855	13	23
					D	0.695	0.255	0.333	1.272
					λ_0^M female	0.005	0.007	0.001	0.024
					λ_0^M male	0.005	0.002	0.002	0.010
					λ_0^R female	0.042	0.016	0.020	0.079
					λ_0^R male	0.015	0.003	0.008	0.021
9	Gen-SS	+	+	–	σ^d female	4.219	0.372	3.651	5.095
					σ^d male	8.105	0.264	7.575	8.606
					N	145	34.206	90	219
					n^{UM}	24	3.286	18	30
					D	0.950	0.223	0.588	1.430

References

1 Beausoleil, R. A., Clark, J. D. & Maletzke, B. T. A long-term evaluation of biopsy darts and DNA to estimate cougar density: an agency-citizen science collaboration. *Wildlife Soc. B.* **40**, 583-592; 10.1002/wsb.675 (2016).

2 Rich, L. N. *et al.* Comparing capture-recapture, mark-resight, and spatial mark-resight models for estimating puma densities via camera traps. *J. Mammal.* **95**, 382-391; 10.1644/13-MAMM-A-126 (2014).

3 Royle, J. A., Chandler, R. B., Sollmann, R. & Gardner, B. Spatial capture-recapture, 577 (Academic Press, 2014).

4 Efford, M. G. *secrdesign: sampling design for spatially explicit capture-recapture*. R package version 2.4.0, https://cran.r-project.org/package=secrdesign/ (2016).

5 Efford, M. G. *secr: spatially explicit capture-recapture models*. R package version 3.0.1, https://cran.r-project.org/package=secr/ (2017).
